# Dynamic endometrial architecture of pregnant fat-tailed dunnarts (*Sminthopsis crassicaudata*)

**DOI:** 10.1530/RAF-25-0113

**Published:** 2025-11-21

**Authors:** Jennifer C Hutchison, Angus H W Sutherland, David L Potter, Sara Ord, Andrew J Pask

**Affiliations:** ^1^ Colossal Australia, School of BioSciences, University of Melbourne, Parkville, Victoria, Australia; ^2^ Hudson Institute of Medical Research, Clayton, Victoria, Australia; ^3^ Department of Molecular and Translational Medicine, Monash University, Clayton, Victoria, Australia; ^4^ Colossal BioSciences, Dallas, Texas, USA

**Keywords:** endometrium, glandular epithelium, marsupial, uterus, pregnancy

## Abstract

**Abstract:**

**In brief:** Extensive remodelling of the fat-tailed dunnart endometrium accompanies gestation, highlighting the importance of endometrial glandular contributions to the marsupial mode of reproduction.

**Abstract:** Eutherian mammal embryos typically implant at the blastocyst stage of development. In contrast, marsupial pregnancy is characterised by a prolonged pre-implantation period and an advanced stage of embryonic development before implantation and placentation. During this early extended phase of pre-implantation growth and development, nutrition is provided predominantly by secretions from the endometrium. Here we histologically examined the fat-tailed dunnart (*Sminthopsis crassicaudata*) endometrium for architectural features, collagen distribution, and blood vessel patterning, as well as characterised the different major cell types. Our data showed significant structural changes across gestation, facilitated by highly proliferative epithelial cells, indicative of a dynamic environment that has evolved to facilitate embryonic development through provision of appropriate nutritional and mechanical cues. The densely glandular endometrium undergoes oedematous expansion across gestation, with sparse stromal cells present in a loose extracellular matrix. Furthermore, our data show outgrowth of complex luminal protrusions containing vascular networks and dense sub-luminal collagen deposits occurring in the peri-implantation stages of development. Concurrently, the luminal epithelium transitions from a pseudostratified columnar epithelium to thin squamous epithelium. Large, elasticated blood vessels are observed throughout the dunnart uterus, with a substantial network of small vessels evident in close proximity to the remodelling luminal epithelium. Our study highlights the fat-tailed dunnart endometrium as a model of endometrial remodelling to explore maternal support of embryonic development. This has implications for the investigation of marsupial maternal–foetal interactions, including histotrophic nutrition and embryo adhesion to the endometrium, opening new doors for comparative and developmental biology.

**Lay summary:**

Understanding how pregnancy works in marsupials is essential to developing new ways to protect some of Australia’s most vulnerable species. Marsupial pregnancy is very short, only 2 weeks in the fat-tailed dunnart, and for most of the pregnancy, the embryo needs nutrients to be provided by the inner lining of the uterus, the endometrium. This work examined structures of the endometrium which support pregnancy in the fat-tailed dunnart. We found an extremely high number of glands, which provide nutrients to the developing embryo, and changes in the surface of the endometrium to allow connection with the embryo, structural support, and the transfer of nutrients and waste. Our study highlights how the fat-tailed dunnart endometrium supports pregnancy in a marsupial, providing foundations for ongoing research into their reproduction. This will ultimately help us develop new conservation tools to support marsupial reproduction.

## Introduction

The ongoing global extinction crisis demands urgent action to protect Earth’s biodiversity. Marsupials in particular face an alarming risk, with up to 40% of Australian species classified as Threatened or Vulnerable to extinction (Woinarski *et al.* 2015, Geyle *et al.* 2018, Woinarski & Fisher 2023). A detailed understanding of marsupial reproductive physiology is paramount to the successful development of next-generation conservation tools to enhance their survival. A major component of marsupial reproduction, which remains highly understudied, is the endometrium: the functional inner layer of the uterus. This research provides descriptions of the endometrial environment and associated histoarchitectural changes which support pregnancy in the fat-tailed dunnart.

A defining characteristic of marsupial reproduction is a short gestation period, followed by birth of highly altricial young and an extended period of postnatal development in the maternal pouch (Renfree 2010, Parrott & Edwards 2023). Despite their altricial state at birth, marsupial embryos are developmentally advanced at the time of implantation. During a prolonged pre-implantation phase, the marsupial embryo is predominantly reliant on histotrophic secretions from the endometrial glands, known to be essential for the establishment of pregnancy in eutherian species (Gray *et al.* 2000, 2002, Whittington *et al.* 2018, Burton *et al.* 2020). Thus, the glandular endometrium and any pregnancy-related variations to its structure and secretions are of key importance to understanding marsupial pregnancy.

In the marsupial endometrium, the stromal compartment is relatively sparse in fibroblastic cells, with a localised compact layer near the luminal epithelium which has a transcriptional signature distinct from eutherian stromal fibroblasts (Laird *et al.* 2016, Basanta *et al.* 2024). In contrast, the glandular epithelium, responsible for secretion of the histotroph, is particularly cell dense (Renfree 1972, Laird *et al.* 2016, Pagliarani *et al.* 2023). Such dense glandular networks have been observed in numerous species including the koala (*Phascolarctos cinereus* (Pagliarani *et al.* 2023)), bettong (*Bettongia giamardi cuniculus* (Flynn 1930)), tammar wallaby (*Macropus eugenii* (Laird *et al.* 2016)), grey short-tailed opossum (*Monodelphis domestica* (Griffith *et al.* 2019)), and the stripe-faced dunnart (*Sminthopsis macroura* (Cruz & Selwood 1997, Menkhorst *et al.* 2009*a*)). A similarly dense glandular epithelium is also observed in monotremes such as the echidna (*Tachyglossus aculeatus* (Fenelon *et al.* 2025)). Detailed comparisons to species such as pigs and ruminants, in which there is an increased reliance on histotrophic nutrition due to extended pre-implantation embryo development and limited trophoblast invasion (Bazer *et al.* 2012, Bazer & Johnson 2014, Kelleher *et al.* 2019, Burton *et al.* 2020, Davenport *et al.* 2023), will determine conserved and marsupial-specific aspects of endometrial glandular biology.

Across gestation, there is an appreciable increase in endometrial thickness (Owen 1834, Flynn 1930, Cruz & Selwood 1993), predominantly due to an oedematous expansion of the extracellular space. Gestation has a mitogenic effect on the endometrial glands, with pulses of proliferation observed at the time of unilaminar blastocyst formation and immediately before implantation in the stripe-faced dunnart (Menkhorst *et al.* 2009*a*), and is further evidenced by increased glandular tissue in the gravid uterus of *Monodelphis domestica* (Griffith *et al.* 2019). While in some marsupials, including the Virginian opossum (*Didelphis virginiana*), the conceptus has limited impact on uterine secretions (Renfree 1975), in others such as the tammar wallaby (*Macropus eugenii*) and the fat-tailed dunnart (*Sminthopsis crassicaudata*), the presence of an embryo influences uterine secretions, including protein concentration and composition which are specific to the stage of gestation (Renfree 1972, 1973, Martin *et al.* 2016, Whittington *et al.* 2018). These gestation-specific changes to glandular morphology and function are proposed to be an indication of endometrial recognition of pregnancy in the opossum (Griffith *et al.* 2019).

Given the diversity of endometrial biology between marsupial species, it is necessary to identify both key characteristics and nuances of endometrial structure and function in emerging model species including the fat-tailed dunnart (*Sminthopsis crassicaudata*), the species of interest in this study. While significant differences exist across marsupial lineages such as macropods vs dasyurids, on an overt and cellular level (Hughes 1974, Laird *et al.* 2017*a*, Parrott & Edwards 2023), they share the common feature of extended pre-implantation development in which concurrent remodelling of the endometrial luminal and glandular epithelium occurs (Cruz & Selwood 1993, Menkhorst *et al.* 2009*a*, Laird *et al.* 2016, 2017*b*, Griffith *et al.* 2019). The timing and degree of implantation of the fat-tailed dunnart embryo, including the development of an endotheliochorial placenta (Roberts & Breed 1994, Newton *et al.* 2025), make it a good model for marsupial reproduction, as many features of its reproduction are representative of the marsupial reproductive strategy.

The fat-tailed dunnart has been thoroughly characterised across embryonic and postnatal development (Cook *et al.* 2021, Newton *et al.* 2025); however, the biophysical and biochemical uterine environment in which these embryos develop remains relatively understudied. We hypothesised that the endometrium of the fat-tailed dunnart has histological characteristics that support the significant glandular contribution to marsupial pregnancy. Here, we present a detailed description of fat-tailed dunnart endometrial biology across gestation to provide baseline data to enhance research into this enigmatic species.

## Materials and methods

### Animal experimentation

All fat-tailed dunnart (*Sminthopsis crassicaudata*) experimentation was approved by the University of Melbourne Ethics Committee (#26863) and performed in alignment with the Australian Code for the Care and Use of Animals for Scientific Purposes. Routinely, fat-tailed dunnarts were housed according to recommended conditions (Scicluna *et al.* 2025), between 21.2 and 22.6°C with a 16 h light:8 h darkness photoperiod to approximate the conditions of ‘summer’ and were given food and water *ad libitum*. A total of 40 fat-tailed dunnarts were examined in this study.

### Timed embryo generation and uterine tissue collection

Sexually mature fat-tailed dunnarts were placed at a 2:1 or 3:1 ratio of females:males and monitored for pregnancy according to previously described methods (Newton *et al.* 2025). Upon detection of the appropriate stage of gestation, fat-tailed dunnarts were humanely killed via cervical dislocation, and the reproductive tract was removed and placed into phosphate-buffered saline (PBS; Gibco, Australia). Uteri were cleared of excess fat and oviductal material and opened to release the embryos, which were staged according to Newton *et al.* 2025. Uteri were rinsed in PBS and fixed in 4% paraformaldehyde at 4°C for 48 h (PFA; Thermo Fisher Scientific, Australia) before processing for paraffin embedding.

### Histological staining

For histological analyses, 5 μm sections were transferred onto Trajan Series 3 microscope slides. Periodic acid-Schiff (PAS), orcein, picrosirius red, and haematoxylin and eosin (H&E) staining were performed as per routine protocols by the Melbourne Histology Platform (University of Melbourne).

### Immunohistochemistry

Tissue sections were deparaffinised in two changes of histolene for 10 min each, then rehydrated in a decreasing gradient of ethanol (2 min in each of: 100, 100, 90, and 70% ethanol (CSA scientific)), followed by 2 min in distilled water. Heat-induced antigen retrieval was performed using 10 mM sodium citrate (pH 6.0; Sigma Aldrich, Australia), whereby sections were covered in citrate buffer and microwaved for 5 min at 750 W, followed by 3 min at 500 W, and then left to stand in the hot buffer at room temperature for 20 min. Sections were then washed three times in TRIS-buffered saline (TBS; 10 mM TRIS, pH 7.6; Sigma Aldrich) for 5 min per wash. Endogenous peroxidase was blocked by application of 3% H_2_O_2_ (Sigma Aldrich) for 5 min at room temperature, then sections were washed in TBS and blocked in 10% normal horse serum (DAKO) + 3% BSA (Sigma Aldrich) in TBS for 1 h at room temperature. They were then incubated with primary antibodies (mouse-anti-vimentin (1:800, Abcam, UK, #ab8069, RRID:AB_306239), mouse-anti-E-cadherin (E-CAD; 1:800; Abcam #ab76055, RRID:AB_1310159), rabbit-anti-cytokeratin 8 (CK8; 1:500; Abcam, China, #ab175249), rabbit-anti-PCNA (1:1,000; Cell Signalling Technologies, Australia, #13110T, RRID:AB_2636979), or rabbit-anti-aquaporin 1 (AQP1; 1:400; Abcam, China, #ab65837, RRID:AB_1141072) diluted in TBS +0.1% BSA overnight at 4°C. Mouse or rabbit IgG was utilised as an isotype control where appropriate (Invitrogen, Australia; E-CAD, VIM, AQP1), TBS +0.1% BSA without primary antibody served as an additional negative control (CK8, PCNA). All negative controls did not produce any signal. Sections were washed, then incubated with biotinylated horse-anti-mouse (1:1,000; Vector Laboratories, USA, #BA-2000-1.5, RRID: AB_2313581) or biotinylated horse-anti-rabbit (1:1,000; Vector Laboratories #BA-1100-1.5, RRID: AB_2336201) as appropriate, for 1 h at room temperature. Antibody binding was visualised using VECTASTAIN ABC-HRP (Vector Laboratories) and 3,3′-diaminobenzidine (DAB; Agilent, USA) as per manufacturers’ instructions. Nuclei were counterstained with Harris’ haematoxylin (Hurstchem).

### Semi-quantification of histological features

To semi-quantify glandular cross-section area, a minimum of ten segments of 500 μm^2^, divided between proximal (approximately 50% of the section closest to the luminal epithelium) and distal (approximately 50% of the section furthest from the luminal epithelium) endometrium, were randomly selected for assessment. Each square was completely occupied by tissue, with no lumen or myometrium encroaching on the section. Cross-sections in each area were manually segmented using QuPath (V0.5.1 (Bankhead *et al.* 2017)), and the median cross-section area was calculated. For one sample, only seven segments were able to be counted. The height of the luminal epithelium was determined by taking the median of 20 measurements perpendicular to the basal membrane of the luminal epithelial cells.

Qualitative scoring was performed for PAS and CK8 staining to approximate staining intensity. CK8 immunodetection was scored from 0 (absent) to 5 (strong) in the luminal and glandular epithelium (proximal and distal), in accordance with prior reports (Wijayarathna *et al.* 2022). Scoring was not performed for E-CAD. Given the lesser degree of colour contrast in a PAS stain, the differentiation of staining intensity could not be performed to the same degree. Hence, we took a three-point scale and categorised staining as weak, moderate, or strong to provide context.

### Visualisation and data analysis

Unless otherwise stated, all images were obtained using an Aperio Pannoramic Scan II and processed using QuPath (V0.5.1 (Bankhead *et al.* 2017)) and ImageJ (Schindelin *et al.* 2012). For analysis, endometrial tissue was classified by the stage of embryonic development and grouped as defined in Table 1. A minimum of three independent biological samples were used for each period of gestation and stain. Statistical analyses and graphical representation were performed using GraphPad Prism V10.3.1 (Boston, Massachusetts, USA). All data were tested for normality using a Shapiro–Wilk normality test. Parametric data were assessed for significance using a one-way ANOVA followed by Tukey’s tests, and non-parametric data were assessed using a Kruskal–Wallis test followed by Dunn’s multiple comparisons. Unless otherwise stated, data are represented as mean ± SEM. Individual dot points represent biologically independent samples.

**Table 1:** Experimental grouping of fat-tailed dunnart endometria across gestation. Endometrial tissue was classified according to the developmental stages of the embryos within the uterus (Newton *et al.* 2025). A minimum of *n* = 3 biologically independent replicates were assessed from each group.

Grouping	Embryonic stages	Embryonic day	Characteristics
Pre-adhesion			
Cleavage	-	0–3	2–16 cells. Presence of deutoplasm
ULB	-	4–8	Unilaminar layer of cells, embryo appears as a hollow sphere
BLB	16–18	9	Pluriblast formation is evident
Neurulation	19–23	10–11	Primitive streak and head folds form. Breakdown of shell coat
Post-adhesion			
Organogenesis	24–27	11–12	Embryo adhesion to the endometrium. Formation of limb buds and organ primordia
Implantation	28–33	13	Embryonic implantation into the endometrium, continued organ development

ULB, unilaminar blastocyst; BLB, bilaminar blastocyst.

## Results

### The fat-tailed dunnart uterus is highly glandular and undergoes gestation-related histological variations

**Figure 1:**
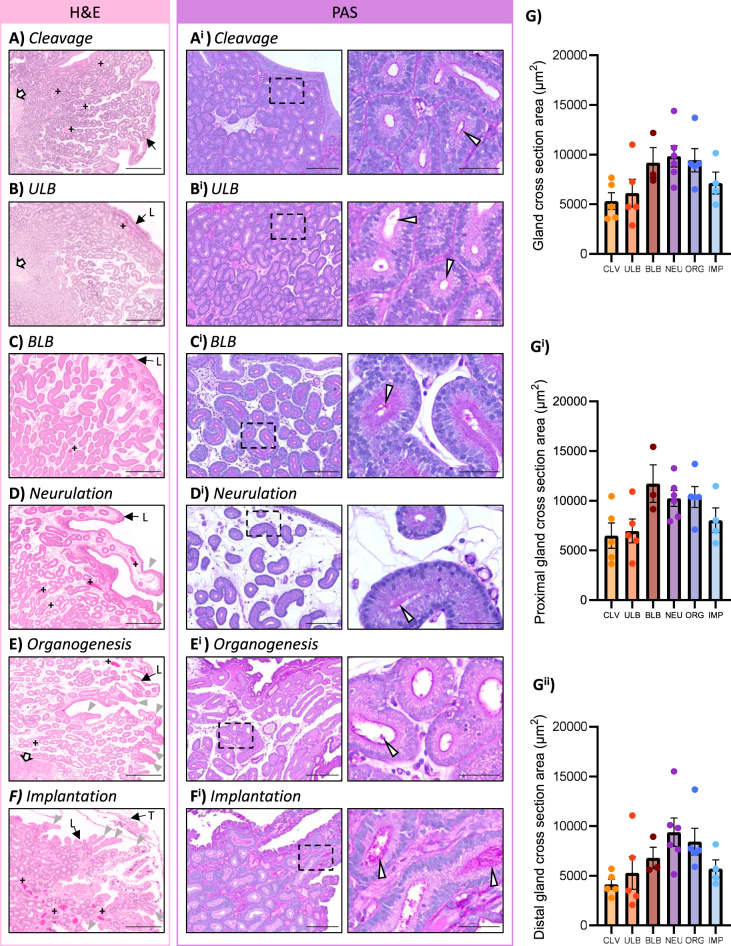
The fat-tailed dunnart endometrium is highly glandular, with increasing oedematous expansion of the stromal compartment across gestation. Representative H&E and PAS-stained endometrial tissue from (A) cleavage (*n* = 5), (B) ULB (*n* = 6), (B) BLB (*n* = 4), (C) neurulation (*n* = 6), (D) organogenesis (*n* = 5), and (E) implantation (*n* = 5) stages of gestation. Black arrow ‘L’ = luminal epithelium, black arrow ‘T’ = trophectoderm, white arrow = myometrium, grey arrowheads = examples of developing CLPs, and ‘+’ = maternal blood vessels. Scale bars represent 500 μm (A, B, C, D, E), and 100 μm (A^i^, B^i^, C^i^, D^i^, E^i^, F^i^). The dashed box indicates the area magnified in the images on the right, in which the scale bar represents 20 μm. The stage of gestation had a significant impact on the average cross-section area of the (G) total glandular epithelium (*P* = 0.0435), (G^i^) glandular epithelium proximal to the luminal epithelium (*P* = 0.0324), and (G^ii^) distal to the luminal epithelium (*P* = 0.0358). CLV, cleavage; ULB, unilaminar blastocyst; BLB, bilaminar blastocyst; NE, neurulation; ORG, organogenesis; IMP, implantation. Statistical tests: one-way ANOVA (G, G^i^) and Kruskal–Wallis (G^ii^).

To analyse the histology of the fat-tailed dunnart endometrium, cross-sections at different stages of gestation from cleavage to implantation were stained with haematoxylin & eosin (Fig. 1A, B, C, D, E, F) and PAS (Fig. 1A^i^, B^i^, C^i^, D^i^, E^i^, F^i^). The uterus is encircled by a smooth muscle layer, the myometrium (Fig. 1A, B, E, white arrow where evident). The endometrium contains a high density of glandular epithelium, few stromal fibroblasts (Fig. 1), and numerous blood vessels (Fig. 1, ‘+’).At the time of embryonic cleavage, the endometrium is relatively compact with a folded surface (Fig. 1A, ‘L’). Minimal PAS + secretions on the apical surface of glandular epithelial cells were observed (Fig. 1A^i^, white arrowheads). This was similar for unilaminar blastocyst (ULB) stage endometria (Fig. 1B and B^i^). By the bilaminar blastocyst (BLB) stage of gestation, the endometrium exhibited regions of expansion (Fig. 1C). Glands had large luminal cross-sections and PAS + accumulations within the gland lumen (Fig. 1C^i^, white arrowheads). By the neurulation stage, the endometrium continued to expand with evidence of elongation of the luminal folds and the development of complex luminal protrusions (Fig. 1D, grey arrowheads). Glandular PAS^+^ secretions were similar to the BLB stage (Fig. 1D^i^, white arrowheads).

Organogenesis represents the developmental period in which fat-tailed dunnart embryos begin to adhere to the endometrium. At this stage, the endometrial tissue had numerous complex luminal protrusions (CLP; Fig. 1E, grey arrowheads), and an oedematous stromal compartment with large blood vessels (Fig. 1E, ‘+’). Apical surfaces of large glandular cross-sections were strongly PAS+ (Fig. 1E). By implantation (Fig. 1F), the stromal compartment appeared more compact. The luminal surface was highly folded with numerous CLPs. Embryonic trophectoderm cells were evident (Fig. 1F, ‘T’) within shallow implantation sites. Strong PAS + secretions were identified within the large lumen of endometrial glands on the apical surface of glandular epithelial cells (Fig. 1F^i^, white arrowheads). In the final stages of gestation (implantation; embryo developmental stage 30+), degeneration of the glands was evident and was accompanied by an increase in cells near the basal surface of the glandular epithelium, which appeared to be of immune origin (Supplementary Fig. 1 (see section on Supplementary materials given at the end of the article)).

To determine if the visually appreciable increase in glandular lumen size was statistically significant, the size of the glandular cross-section was quantified as a proxy for the lumen given the small nature of some glands. Quantification detected a significant effect of gestational stage on the size of glandular cross-sections (*P* = 0.0435; Fig. 1G); however, post hoc analysis could not detect a stage-specific effect (*P* ≥ 0.0754 for all comparisons). This trend was conserved both in the proximal (overall *P* = 0.0324, *P* ≥ 0.0893 for all comparisons; Fig. 1G^i^) and distal (overall *P* = 0.0358, *P* ≥ 0.0589 for all comparisons; Fig. 1G^ii^) glandular epithelium.

### The luminal epithelium undergoes an EMT-like transformation

**Figure 2:**
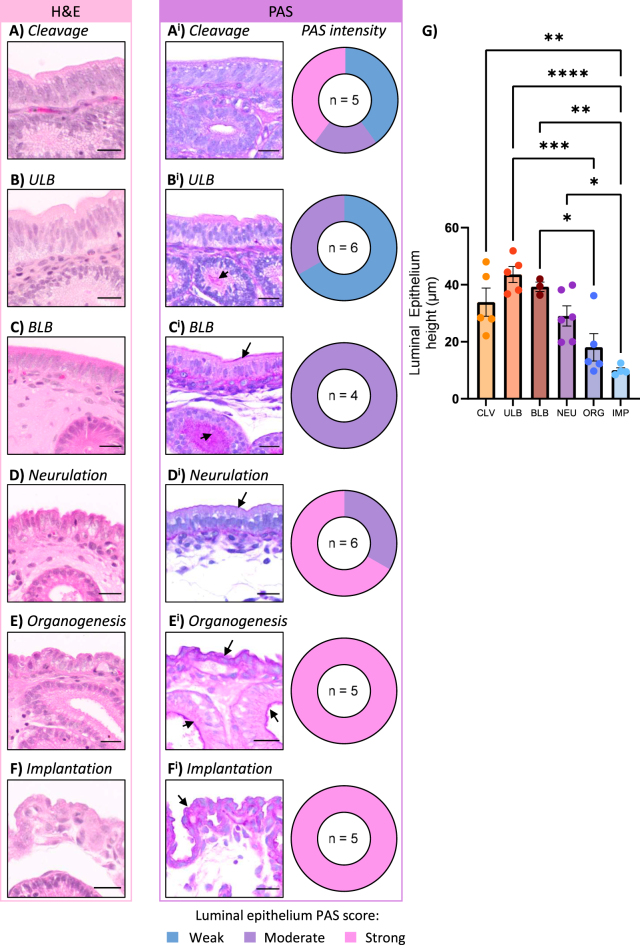
The luminal epithelium progressively loses polarity during gestation. Representative images of H&E and PAS-stained luminal epithelium from (A) cleavage (*n* = 5), (B) ULB (*n* = 6), (C) BLB (*n* = 4), (D) neurulation (*n* = 6), (E) organogenesis (*n* = 5), and (F) implantation (*n* = 5) stages of gestation. (G) The luminal epithelium was significantly reduced in height at organogenesis and implantation compared to the preceding stages. The black arrow indicates PAS-positive accumulations. Pie charts represent visual scoring of PAS intensity observed on the luminal epithelium: weak (blue), moderate (purple), and strong (pink). Scale bars represent 25 μm. CLV, cleavage; ULB, unilaminar blastocyst; BLB, bilaminar blastocyst; NEU, neurulation; ORG, organogenesis; IMP, implantation. *P < 0.05, **P < 0.01, ***P < 0.001, ****P <0.0001. Statistical test: one-way ANOVA followed by Tukey’s multiple comparisons.

To identify overt morphological changes associated with endometrial implantation and the reduction in luminal epithelial height, the luminal epithelium was measured from basal to apical membrane, and PAS+ secretions on the luminal surface were visually scored as weak (Fig. 2, blue in pie charts), moderate (Fig. 2, purple in pie charts), and strong (Fig. 2, pink in pie charts).

During cleavage stages of development, the luminal epithelium was pseudostratified with a high density of ovoid nuclei and a smooth apical surface (Fig. 2A). The intensity of PAS+ accumulations at the apical surface of luminal epithelium varied, with samples showing weak, moderate, or strong accumulations (Fig. 2A^i^). During the ULB stage of gestation, the luminal epithelium was similar to the preceding stage, with a pseudostratified layer of epithelial cells and densely packed ovoid nuclei (Fig. 2B) and weak to moderate PAS+ secretions (Fig. 2B^i^). By the BLB stage of gestation, most nuclei were lining up next to each other, forming a simple columnar epithelium (Fig. 2C). In all sections examined, the luminal epithelium had moderate levels of PAS+ accumulations (Fig. 2C^i^). The luminal epithelium at the stage of neurulation had a rougher surface indicative of breakdown in cell–cell junction integrity (Fig. 2D). Most sections examined had strong PAS+ accumulations on the apical surface of the luminal epithelium (Fig. 2D^i^). By organogenesis (Fig. 2E), the luminal epithelium was strongly PAS+ and was significantly reduced in height compared to ULB and BLB stage luminal epithelium (*P* < 0.01, *P* = 0.0203 respectively; Fig. 2G). By implantation stages (Fig. 2F), the luminal epithelium was significantly reduced in height compared to all preceding stages apart from organogenesis (*P* < 0.01 vs cleavage, *P* < 0.0001 vs ULB, *P* < 0.01 vs BLB, *P* = 0.0181 vs neurulation, *P* = 0.6932 vs organogenesis; Fig. 2G). The epithelium appeared squamous, had less obvious polarity, and cells were strongly PAS+ (Fig. 2E^i^).

### Multiple glandular morphologies are observed in the endometrium

**Figure 3:**
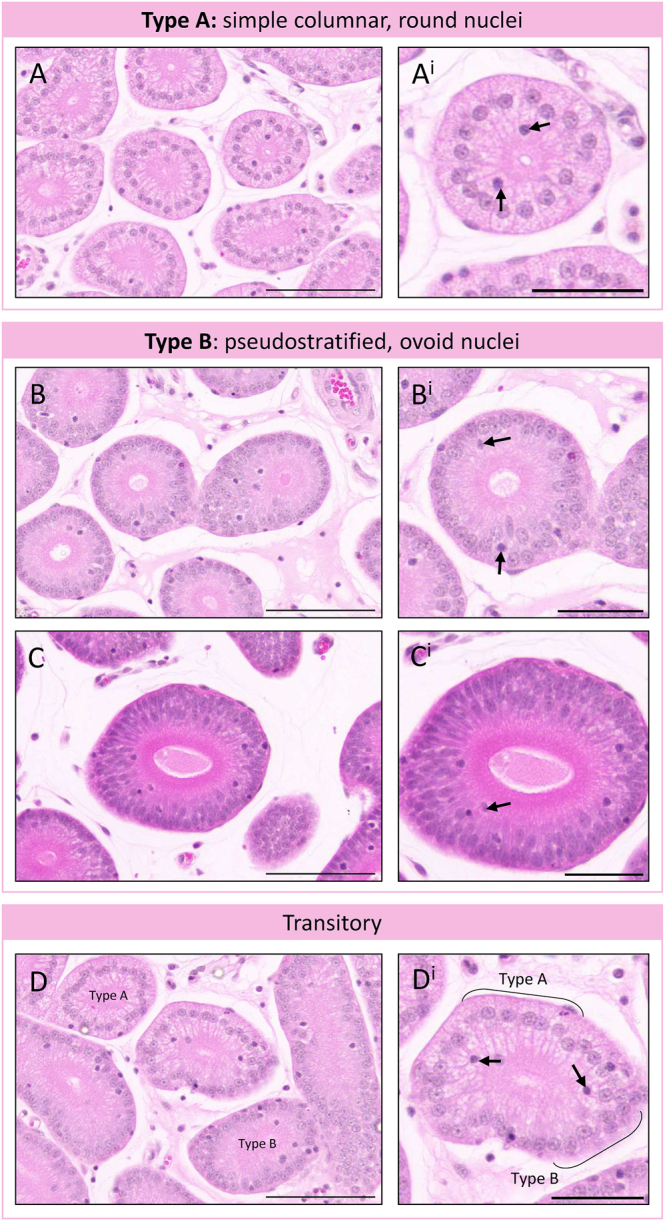
Glandular morphology in the fat-tailed dunnart endometrium. Within the same section of endometrium, glandular morphologies can be classified as simple columnar type A (A, A^i^) and a more polarised, pseudostratified type B (B, B^i^), which can have multiple layers of nuclei (C, C^i^). Some gland cross-sections show a mixture of phenotypes, indicated by brackets (D, D^i^). The black arrow represents nuclei of cells apparently traversing the epithelium. Scale bars represent 100 μm (A, B, C, D), or 50 μm (A^i^, B^i^, C^i^, D^i^).

Closer examination of the endometrial glands revealed two types of glandular cross-sections within individual tissue sections of the endometrium (Fig. 3). Type A glandular cross-sections appeared to have a simple columnar epithelial morphology with predominantly centrally located, round nuclei (Fig. 3A and A^i^). Type B glandular cross-sections appeared to have a pseudostratified epithelium with basally located, ovoid nuclei and obvious apicobasal polarity (Fig. 3B and B^i^). In some cases, type B glands had numerous layers of nuclei in a pseudostratified epithelium (Fig. 3C and C^i^). In some glandular cross-sections, the epithelium showed a mix of morphologies (Fig. 3D), indicating that these glands are in a transitory state. Cells traversing the epithelial barrier were observed in all types of glands (Fig. 3, black arrows) and may be immune cells.

The open, highly secretory glands of the uterus post-neurulation mainly appeared to be of type B morphology; however, a small number of type A glands were evident. While the luminal area of a large proportion of type A glands predominantly appeared very small, both types of glands had PAS+ secretions accumulated on the luminal surface of the epithelium, indicating that they were active.

### Neurulation is associated with genesis of complex luminal protrusions from the endometrium

**Figure 4:**
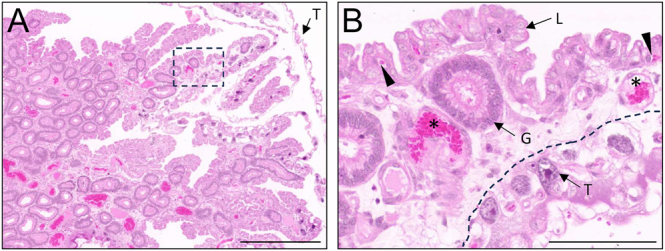
Complex luminal protrusions provide attachment zones for trophectoderm. (A) H&E-stained implantation-stage uterus showing developed complex protrusions from the luminal surface of the endometrium. Luminal folds are lined with luminal epithelium (L), contain secretory glandular epithelium (G), large blood vessels (*), and smaller blood vessels subjacent to the luminal epithelium (black arrowhead). In some regions, the trophectoderm (T) has eroded through the luminal epithelium. Scale bars represent 500 μm (A) and 100 μm (B). The black dotted box indicates the enlarged region presented in B. The dotted line in B represents the border of the maternal–foetal interface.

From approximately neurulation, the folded luminal surface of the endometrium begins to develop complex luminal protrusions (CLP; Fig. 1D, E, F). These appear to both increase the surface area of the endometrium and provide attachment surfaces for the developing embryo (Fig. 4A and B). These highly complex extensions of the luminal surface contain secretory glands, blood vessels of varying sizes, and are lined by a cobblestone luminal epithelium. In some areas, trophectoderm cells from the avascular, bilaminar yolk sac are observed to have eroded the luminal surface.

### Epithelial identity markers fluctuate throughout the endometrium across gestation

**Figure 5:**
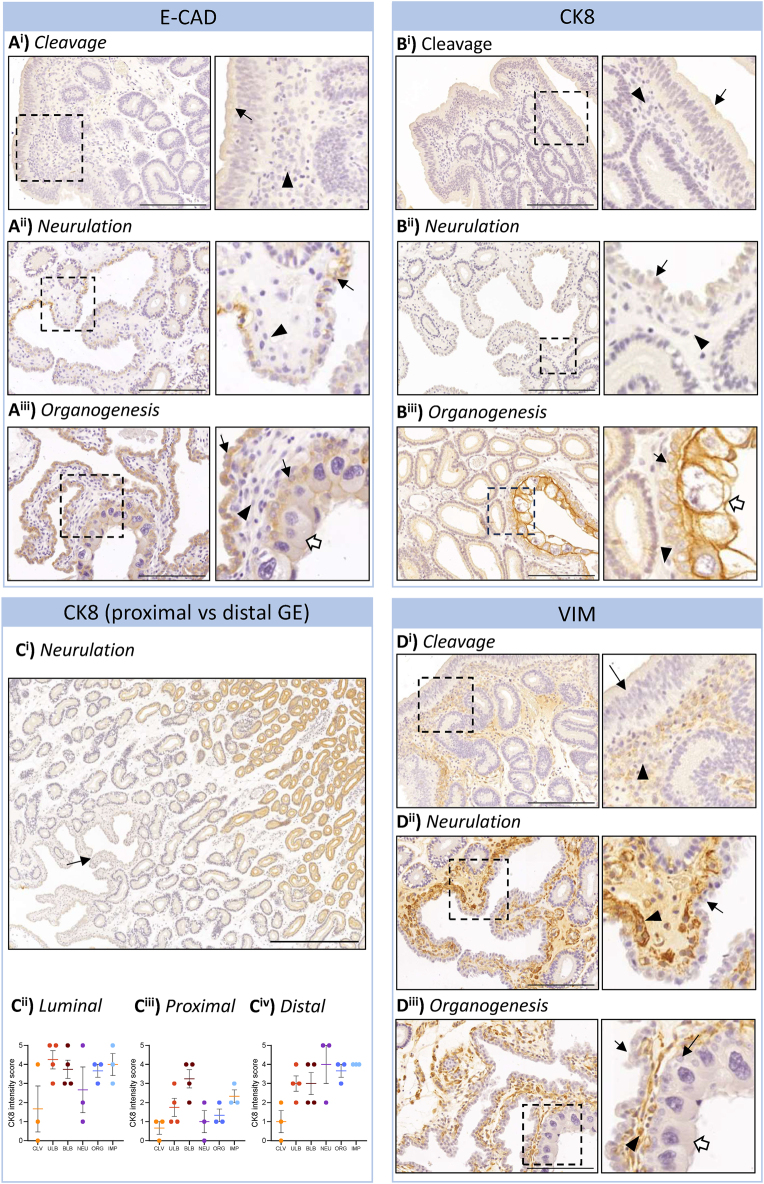
Epithelial and stromal identity of endometrial cells is confirmed by immunoreactivity. The cellular identity of the surface and glandular lining appears morphologically epithelial. (A) E-CAD immunoreactivity confirms epithelial identity in the luminal epithelium in the cleavage (A^i^), neurulation (A^ii^), and organogenesis (A^iii^) stages of gestation. Immunoreactivity was weak in early stages of gestation, increasing in intensity of detection from neurulation. Embryonic trophectoderm are E-CAD+. (B) CK8 followed the same staining pattern in the luminal epithelium as E-CAD across gestation. Representative images are shown from cleavage (B^i^), neurulation (B^ii^), and organogenesis (B^iii^) stages of gestation. (C) In the glandular epithelium, CK8 was most strongly detected in the glandular epithelium distal to the luminal epithelium (C^i^). No significant difference in intensity was detected between stages of gestation in the luminal epithelium (*P* = 0.4515; C^ii^) or distal glandular epithelium (*P* = 0.0904; C^iv^). An overall effect of gestational stage was detected in the proximal glandular epithelium (*P* = 0.0386), but no significant difference was detected between groups (*P* ≥ 0.0556 for all comparisons). *n* ≥ 3 for all. Black arrow = luminal epithelium, black arrowhead = stromal cells, white arrow = trophectoderm. The dotted box indicates the enlarged region. Scale bars represent 200 μm (A, B, D) or 500 μm (C). Negative controls are provided in Supplementary Fig. 2. CLV, cleavage; ULB, unilaminar blastocyst; BLB, bilaminar blastocyst; NEU, neurulation; ORG, organogenesis; IMP, implantation. Statistical tests: Kruskal–Wallis followed by Dunn’s multiple comparisons test.

To confirm the cellular identity of major endometrial features, immunohistochemistry for E-Cadherin (E-CAD) and cytokeratin 8 (CK8) was performed. E-CAD expression was moderately low in the cleavage and neurulation stage endometrium (Fig. 5A^i^ and A^ii^). By organogenesis (Fig. 5A^iii^), E-CAD was strongly detected in the luminal epithelium and was present in the trophectoderm. CK8 was localised to the luminal and glandular epithelium and the trophectoderm (Fig. 5B^i,ii,iii^). CK8 was more strongly expressed in the trophectoderm compared to the glandular epithelium. The population of glands proximal to the luminal epithelium had lower CK8 expression than glands more distal to the luminal epithelium (Fig. 5C^i,ii,iii,iv^). There was no significant difference in luminal epithelium CK8 intensity across gestation (*P* = 0.4515; Fig. 5C^ii^) nor in the distal glandular epithelium (*P* = 0.0904; Fig. 5C^iv^). In the proximal glandular epithelium, a significant effect of gestational stage was detected (*P* = 0.0386; Fig. 5C^iii^), but no group-specific effect was detected (*P* ≥ 0.0556 for all comparisons).

### Stromal fibroblasts are localised in proximity to epithelial structures

To confirm the identity of the cell layer immediately below the luminal epithelial cells and in the compartment surrounding the epithelial glands, vimentin (VIM) immunohistochemistry was performed (Fig. 5D). VIM+ cells were predominantly located in proximity to epithelial cell structures (luminal and glandular epithelium). Endothelial cells were also VIM+.

### Epithelial cells are highly proliferative throughout gestation

**Figure 6:**
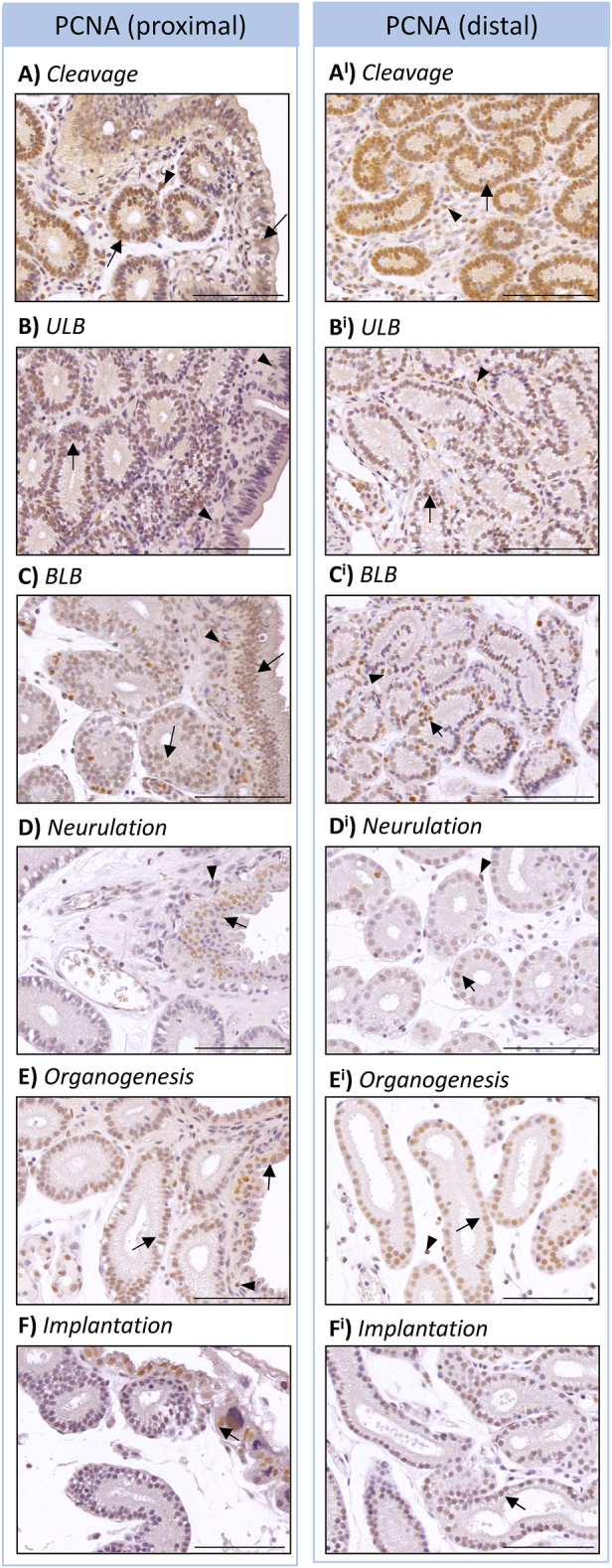
Endometrial epithelial cells are highly proliferative. Nuclear PCNA is evident in the glandular and luminal epithelium from (A) cleavage (*n* = 3), (B) ULB (*n* = 4), (C) BLB (*n* = 4), (D) neurulation (*n* = 3), (E) organogenesis (*n* = 4), and (F) implantation (*n* = 3) stages of gestation. There was no overt difference in PCNA intensity between epithelium proximal (A, B, C, D, E, F) or distal (A^i^, B^i^, C^i^, D^i^, E^i^, F^i^) to the luminal epithelium. Scale bars represent 200 μm. Black arrows indicate positive staining in the epithelium. Black arrowheads indicate PCNA+ cells. Negative controls are provided in Supplementary Fig. 3.

To identify which cells are proliferating and likely to contribute to the development of architectural features such as the CLPs, PCNA immunohistochemistry was performed on endometrial sections (Fig. 6). The number of PCNA+ cells in the glandular epithelium, both proximal and distal to the luminal epithelium, appeared greatest in cleavage and ULB stages of gestation (Fig. 6A and B). At the neurulation stage, very few PCNA+ cells were detected (Fig. 6D), while this number increased again during organogenesis (Fig. 6E). Finally, during implantation, more proliferating cells were detected in the epithelium of distal endometrial glands compared to proximal glands (Fig. 6F and F^i^).

### Endometrial expansion occurs without a visually overt increase in collagen

**Figure 7:**
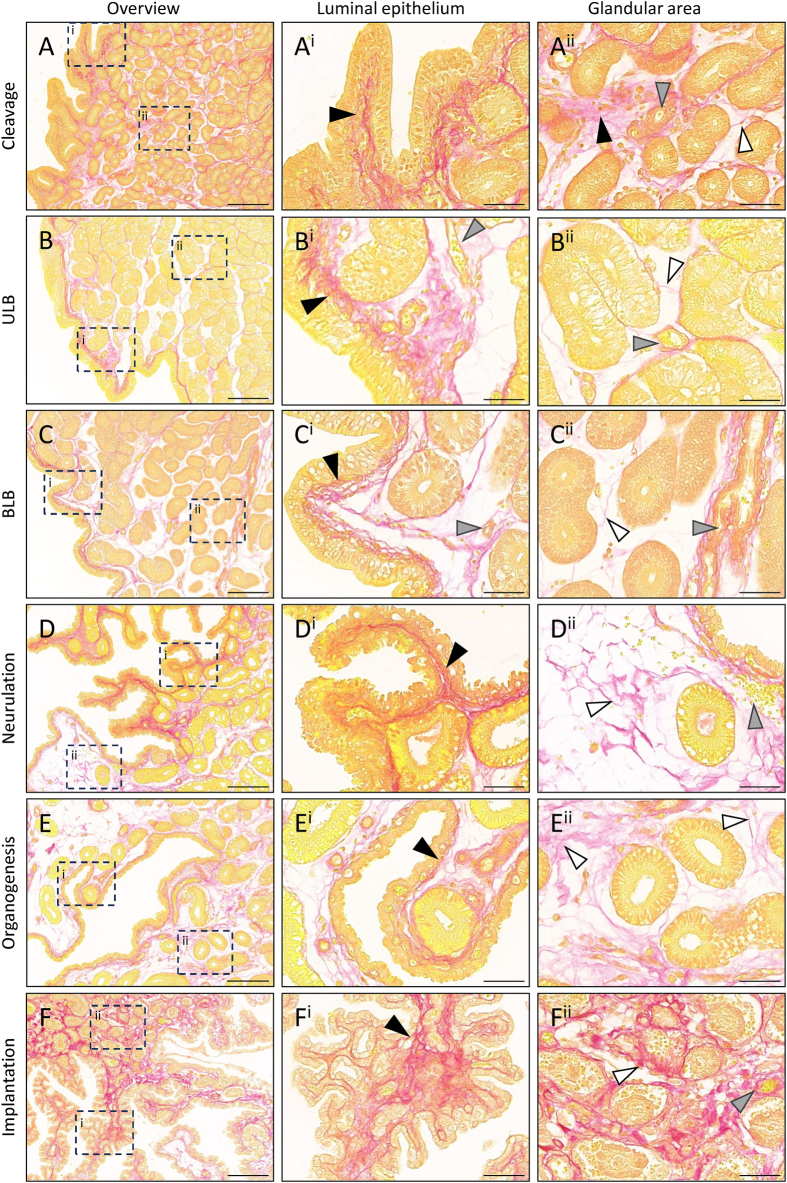
Loosely packed collagen fibrils accommodate endometrial expansion during gestation. Collagen fibrils are detected throughout the endometrium at (A) cleavage (*n* = 3), (B) ULB (*n* = 4), (C) BLB (*n* = 4), (D) neurulation (*n* = 3), (E) organogenesis (*n* = 3), and (F) implantation (*n* = 4) stages of gestation. Collagen is densely packed subjacent to the luminal epithelium (A^i^ – F^i^) and more loosely distributed throughout the glandular region (A^ii^ – F^ii^). Dotted boxes indicate enlarged regions. Black arrowhead = basal side of luminal epithelium, grey arrowhead = maternal blood vessel, white arrowhead = thin collagen fibrils. Scale bars represent 200 μm (A-F) or 50 μm (A^i^ – F^i^ and A^ii^ – F^ii^).

To examine the ECM structure surrounding the glands, picrosirius red staining was utilised to identify collagen fibrils across different stages. Collagen fibrils were strongly detected in the endometrial ECM at all stages of gestation (Fig. 7). During the cleavage stage, fibrils were abundant throughout the compact endometrial compartment and basal to the luminal epithelium (Fig. 7A).

Similarly, during the ULB stage of gestation, collagen fibrils were clearly evident subjacent to the luminal epithelium (Fig. 7B). In contrast, in the expanded regions of the glandular endometrium, collagen fibrils appeared dispersed, indicating limited ECM deposition in the glandular endometrium during expansion (Fig. 7B). This was similar at the bilaminar blastocyst (Fig. 7C), neurulation (Fig. 7D), and organogenesis (Fig. 7E) stages of gestation. Collagen fibrils were also strongly detected in nascent complex luminal protrusions (Fig. 7D^i^). By the time of implantation and close to parturition (Fig. 7F), collagen deposits remained strongly evident throughout the complex luminal protrusions (Fig. 7F^i^) and throughout the glandular compartment (Fig. 7F^ii^). This may be due to shrinkage of this compartment in preparation for birth and post-partum remodelling. Throughout gestation, larger maternal blood vessels were commonly observed surrounded by strong collagen depositions (Fig. 7A, B, C, D, F, grey arrows).

### Vascularisation reveals the intensive blood circulation of the uterus

**Figure 8:**
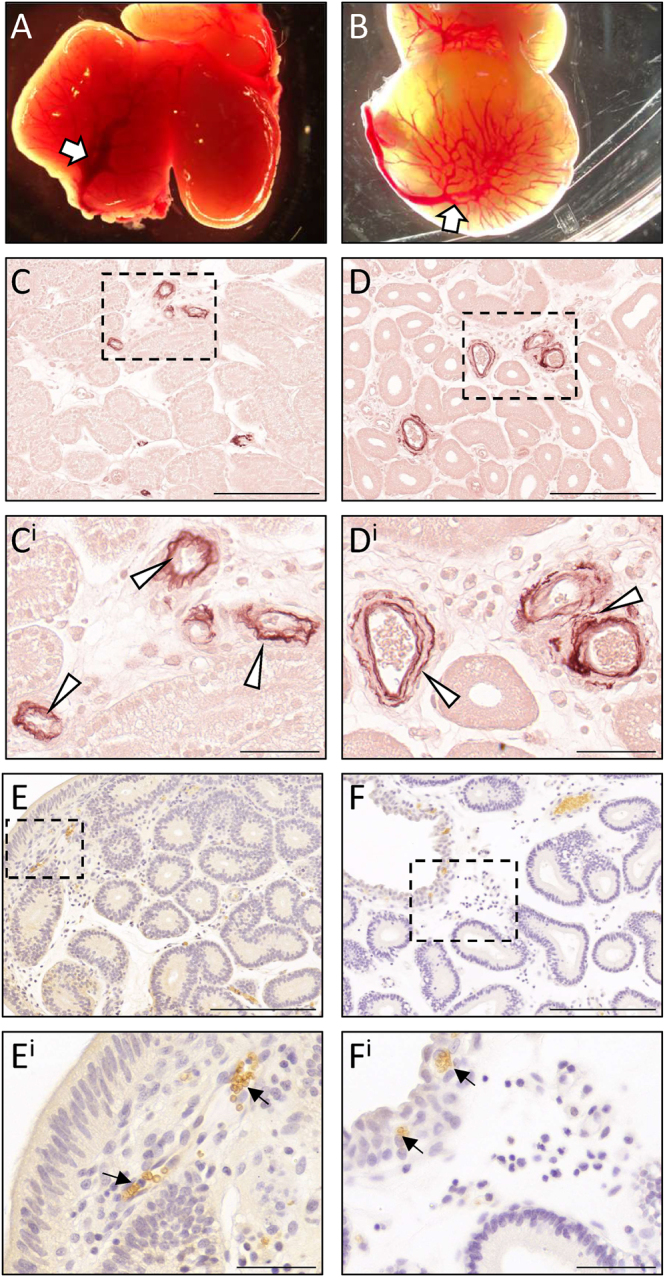
Blood vessels of the fat-tailed dunnart endometrium before and after embryo adhesion. Representative images of dissected uteri (A) before and (B) following embryo adhesion to the endometrium show large uterine vessels on the surface of the uterus. Large orcein+ elasticated blood vessels are found deep within the endometrium both (C) pre- and (D) post-adhesion. Orcein-negative vessels containing AQP1+ red blood cells are found in varying sizes throughout the endometrium and close to the luminal epithelium both (E) pre- and (F) post-adhesion. White arrows = large uterine vessels, white arrowheads = orcein+ blood vessels, black arrows = AQP1+ red blood cells. Scale bars represent 200 μm (C, D, E, F) and 50 μm (C^i^, D^i^, E^i^, F^i^). Negative controls are provided in Supplementary Fig. 4.

To identify elasticated blood vessels (arteries and veins) and capillary networks, orcein staining and aquaporin 1 (AQP1) immunohistochemistry were performed. The vasculature of the uterus was very similar at different stages before implantation; hence, we compared the vasculature between pre- and post-adhesion (Fig. 8). At a gross morphological level, large blood vessels could be seen attached to the surface of the fat-tailed dunnart uterus at all stages (Fig. 8A and B). Within the endometrium, large elasticated, orcein+ blood vessels were seen in regions distal to the luminal epithelium. No visually obvious change to the size and distribution of these was evident across gestation (Fig. 8C and D), and multiple layers of elastic fibres were present (Fig. 8C^i^ and D^i^, white arrowheads). Many blood vessels of varying sizes were distributed throughout the endometrium, identified by the AQP1+ blood cells contained within (Fig. 8E and F). Based on their location and in comparison with Fig. 8C and D, these are likely to be orcein-negative. Both pre- and post-adhesion, small clusters of AQP1+ red blood cells were visible immediately beneath the luminal epithelium, indicative of a subepithelial capillary network (Fig. 8E^i^ and F^i^, black arrows).

## Discussion

Plasticity of the uterine environment is essential to accommodate pregnancy; understanding this environment is of central importance to both improving assisted reproduction technologies and translating standardised laboratory techniques from eutherian to marsupial species. This research provides the first baseline data on healthy endometrial biology across gestation of the fat-tailed dunnart (*Sminthopsis crassicaudata*) and highlights several key features: a high density of endometrial glands, dynamic changes to the luminal epithelium, and the formation of complex luminal protrusions. These features are likely integral to supporting embryo development, adhesion, and implantation, thus demonstrating that the fat-tailed dunnart has evolved extensive adaptations to optimise the efficiency of maternal–foetal communication in a histotrophic-centric reproductive strategy.

The glandular network of the fat-tailed dunnart endometrium is exceptionally dense, with compacted stroma only evident at the early stages of gestation (Fig. 1). The relatively low proportion of stromal fibroblasts tends to line epithelial structures, with expansion of the endometrium likely to be attributable to oedema rather than fibroblast proliferation or collagen deposition, which is supported by the transcriptional profile of marsupial stromal fibroblasts that shows lower expression of proliferation markers (e.g. *MKI67* and *TOP2A*) compared to rodents (Basanta *et al.* 2024). Glandular density appears to be similar between marsupials of differing body size and litter number (Menkhorst *et al.* 2009*a*, Laird *et al.* 2016, Griffith *et al.* 2019, Pagliarani *et al.* 2023). This comparatively high proportion of glands compared to eutherian endometria highlights the role of histotrophic nutrition in the extended pre-implantation phase of marsupial gestation. The similarities between the endometrial structure and adaptations to pregnancy of the fat-tailed dunnart and other species with non-invasive placentation, such as ruminants and pigs, reinforce the conserved role of histotrophic nutrition in embryonic development (Bazer *et al.* 2012, Bazer & Johnson 2014, Johnson *et al.* 2018, Davenport *et al.* 2023).

In this study, multiple morphologies were identified within gland cross sections (Fig. 3). It could be hypothesised that they play differential roles in the independent secretion of the uterine histotroph and shell coat proteins, which are essential for normal marsupial embryonic development (Hughes 1974, Casey & Selwood 2003, Menkhorst *et al.* 2008, Frankenberg & Renfree 2018). However, this is unlikely to be the case for two reasons. First, hormonally modulated components of the fat-tailed dunnart shell coat have been immunolocalised to the glandular epithelium of the oviductal isthmus and become progressively restricted to the luminal epithelium and peri-luminal glandular epithelium (Roberts *et al.* 1994, Menkhorst *et al.* 2009*b*). Second, shell coat glands in other species, such as the echidna, have a distinct morphology (Fenelon *et al.* 2025), and this more overt phenotype may be due to their oviparity requiring increased production of shell constituents. Rather, the presence of intermediate morphologies implies maturation along the length of an individual glandular process, as multiple cross sections may be taken from a single gland within a tissue section given the tortuous nature of endometrial glands, or the presence of a basalis gland region similar to the koala endometrium (Pagliarani *et al.* 2023), a theory supported by the gradient of CK8 in the glandular epithelium. Single-cell RNA sequencing of the endometrium will be required to clarify the relevance of these morphologies and epithelial marker intensities to glandular maturation and function.

The fat-tailed dunnart luminal epithelium undergoes dramatic remodelling reminiscent of epithelial–mesenchymal transition (EMT; Fig. 2), similar to the reduction in luminal epithelium height reported in *Antechinus stuartii* (Cruz & Selwood 1993). This process may be mediated by rearrangement of cell–cell adhesions in the fat-tailed dunnart epithelium (Laird *et al.* 2017*c*, Dudley *et al.* 2017, Whittington *et al.* 2018, Buddle *et al.* 2019), and the reduced number of nuclei may be further explained by the holocrine secretion of the shell coat, which necessitates the replacement of epithelial cells (Casey *et al.* 2002). Such a reduction in polarity is necessary for endometrial receptivity due, at least in part, to the mutual repulsion of polarised surfaces (Whitby *et al.* 2018, 2020). Coinciding with the timing of shell coat breakdown in the latter stages of neurulation (Hughes 1974), this morphological transition of the luminal epithelium likely facilitates embryo-endometrium adhesion and trophectoderm invasion. Further studies are warranted to examine how these structural changes relate to embryo implantation and the establishment of the endotheliochorial placenta of the fat-tailed dunnart (Roberts & Breed 1994).

The progression of gestation is accompanied by evagination of complex luminal protrusions (CLPs), with implantation sites evident in discrete regions of CLPs reminiscent of adhesion zones in the tammar wallaby (*Macropus eugenii*) (Ager *et al.* 2007). While bilaminar blastocysts have previously been reported within pockets of the endometrium (Roberts & Breed 1994), CLPs were only histologically evident from the time of neurulation in the sections examined here (Fig. 1). Given the oedematous nature of the stromal compartment, the early embryo may embed into an existing fold through mechanical pressure before expansion rather than being enveloped by an outgrowing CLP. In early pregnancy, folds of the endometrial surface are less evident, which is potentially required for sperm transport and positioning of the fertilised zygotes within the uterus, similar to what has been described in mice (Madhavan *et al.* 2022).

Functionally, CLPs may play similar roles to implantation crypts. In addition to driving the appropriate uterus–embryo axis, the growth of CLPs around the embryos may facilitate paracrine exchange between maternal and foetal tissues through the proximity of the endometrial glandular and vascular network. Coupled with the large blood vessels detected throughout, the identification of numerous blood vessels in CLPs highlights the need for blood transportation and oxygenation throughout the large fat-tailed dunnart endometrium, with the luminal capillary network providing an efficient mechanism for nutrient and gas exchange and providing an interesting comparison to the placentomal and interplacentomal regions observed during sheep placentation (Johnson *et al.* 2018). In addition, CLPs provide an increased endometrial surface area available for implantation; in the tammar wallaby, the endometrial surface area increases up to 2.75-fold in the gravid uterus (Muenzenberger *et al.* 2025). Furthermore, the physical presence of CLPs may enhance mechano-transduced signals akin to the effect of endometrial folds in porcine placentation (Seo *et al.* 2020). Fat-tailed dunnart CLPs are collagen-rich, which may further control the localisation and depth of trophectoderm invasion, as both mechanical stiffness and ECM deposition are known regulators of trophoblast invasion (Kaloglu & Onarlioglu 2010, Cao *et al.* 2023).

While the dunnart uterine environment is highly responsive to steroid hormones (Menkhorst *et al.* 2009*a*,*b*, Dudley *et al.* 2019), the cellular processes orchestrating marsupial endometrial remodelling have not yet been fully elucidated. Our detection of a cell population near degenerating glands close to parturition, which is likely to comprise macrophages and uterine natural killer cells (Díaz-Hernández *et al.* 2021), indicates contribution of the immune system to endometrial remodelling in the fat-tailed dunnart, including tissue resorption in preparation for parturition. It has been proposed that post-partum remodelling of the endometrium is partially mediated by immune cell breakdown of polysaccharide constituents and the subsequent extrusion of macrophages through the glandular epithelium, which in turn promotes the loss of water from the stromal compartment (Shorey & Hughes 1972, Padykula & Taylor 1976). In menstrual species, the endometrial stratum functionalis is cyclically shed and regenerated from a stem-cell-rich basal layer, the stratum basalis (Salamonsen *et al.* 2021, Tempest *et al.* 2022). Given the substantial growth of complex luminal protrusions, it would also be of significant interest to identify the endometrial stem cell contingent contributing to tissue maintenance and remodelling and their potential provision of the highly proliferative cells observed in the luminal and glandular epithelium.

Our histological analyses have revealed the highly glandular nature of the fat-tailed dunnart endometrium, reflective of the importance of histotrophic nutrition in this species. While bounded by the inherent limitations of histology, comparison to other marsupial species revealed a similarly high density of glandular epithelium and striking similarities in luminal epithelium changes, lending credence to our findings. This research has implications for comparative evolutionary and developmental biology; it advances our understanding of marsupial pregnancy and early control of marsupial development, with broader relevance to the evolution of viviparity and the transition from marsupial to eutherian reproductive strategies. Finally, functional clues gleaned from examination of the fat-tailed dunnart endometrium will inform the development of *ex vivo* embryo culture, a likely core technology of the next generation of conservation strategies required to stem the tide of marsupial extinction.

## Supplementary materials



## Declaration of interest

Additional research and salary funding was provided by Colossal BioSciences (Texas, USA).

## Funding

This work was funded by generous philanthropic donations from the Wilson Family Trust and supported by Colossal BioSciences USA.

## Author contribution statement

JCH and AJP conceived the experimental design. JCH performed experiments, data analyses, and wrote the manuscript. AHWS performed experiments and contributed to data analysis. DLP provided critical discussion regarding experimental results. SO facilitated experiments and AJP provided critical discussion regarding results and assisted with manuscript preparation. All authors reviewed the manuscript and provided editorial feedback to JCH.

## Data availability

The data underlying this article will be shared on reasonable request to the corresponding author.
