# Supplementary material for: Dynamic endometrial architecture of pregnant fat-tailed dunnarts (Sminthopsis crassicaudata)
Source: Reprod Fertil. 2025 Nov 21;6(4):e250113. doi: 10.1530/RAF-25-0113 (PMC12641594; doi:10.1530/RAF-25-0113)
Supplement: Supplementary file 1 [file supplementary_materials.pdf]

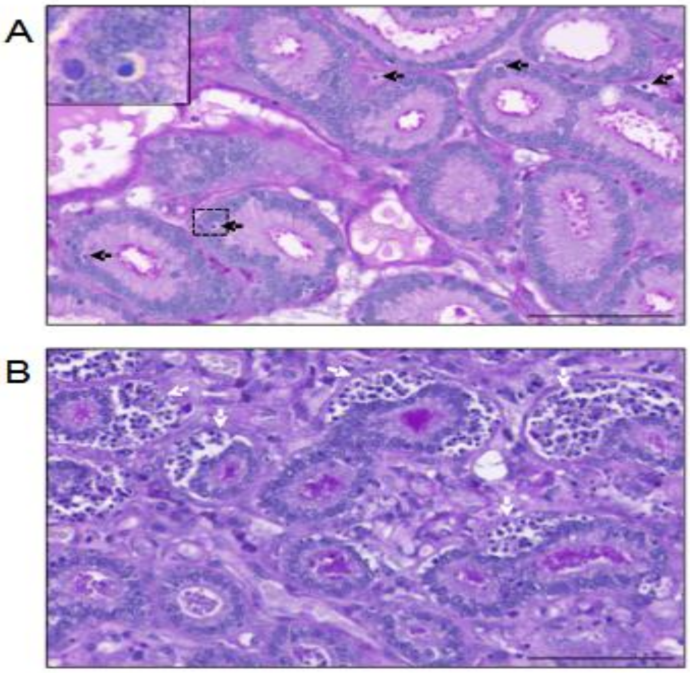

Supplementary Figure 1: Degeneration of epithelial glands in the peri-parturition endometrium. PAS-stained endometria from uteri containing **A)** stage 31 and **B)** stage 32 (of 33 stages) embryos. In A, cells with clear cytoplasm and large round nuclei are evident (black arrow). In B, these cells are numerous, and the glands appear to be shrinking away from the basement membrane (white arrow). Dashed box indicates enlarged region in A. Scale bar represents 100 µm.

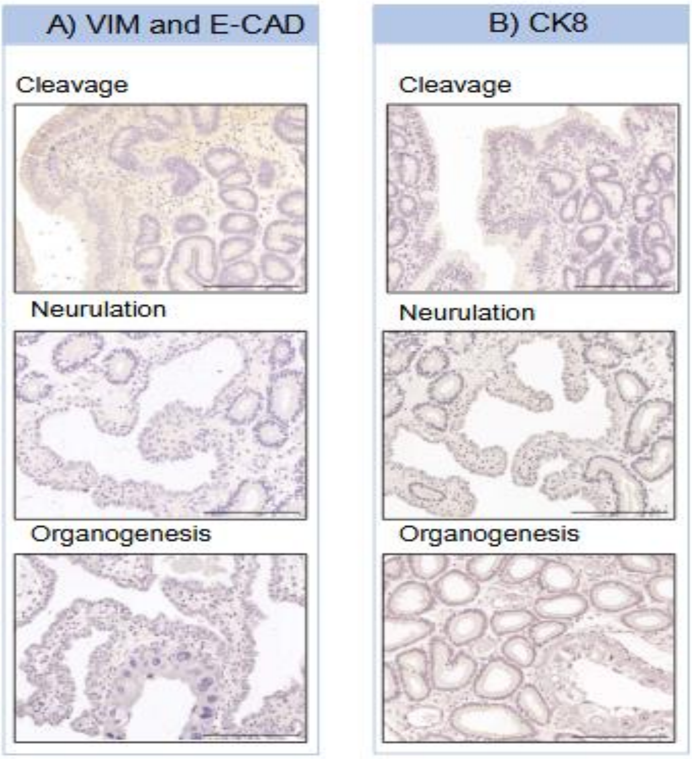

Supplementary Figure 2: Controls for immunohistochemistry presented in Figure 5. A) Negative controls (IgG isotype) pertaining to VIM and E-CAD where immunostaining was performed during the same experiment. B) Negative control (no antibody) for CK8 immunostaining. Scale bars represent 200 µm.

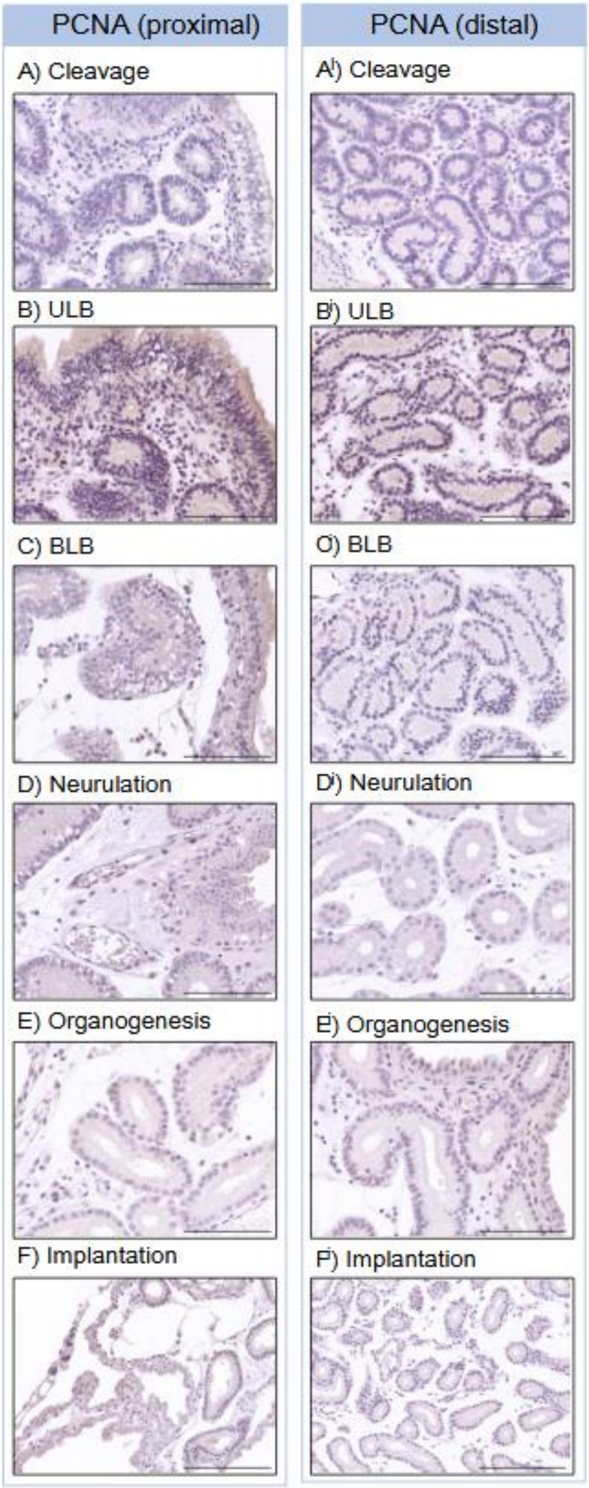

Supplementary Figure 3: Controls for immunohistochemistry presented in Figure 6. Negative controls (no antibody) pertaining to PCNA immunolocalization presented in Figure 6. Scale bar represents 200 µm.

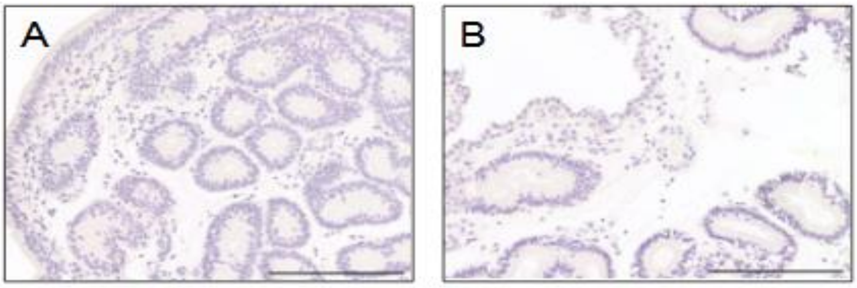

Supplementary Figure 4: Controls for immunohistochemistry presented in Figure 8. Negative controls (IgG isotype) pertaining to AQP1 immunolocalization presented in Figure 8E **(A)** and 8F **(B)**. Scale bar represents 200 µm.
